# Negative regulation of melatonin secretion by melatonin receptors in ovine pinealocytes

**DOI:** 10.1371/journal.pone.0255249

**Published:** 2021-07-29

**Authors:** Julie Lépinay, Catherine Taragnat, Jean-Philippe Dubois, Didier Chesneau, Ralf Jockers, Philippe Delagrange, Véronique Bozon

**Affiliations:** 1 Physiologie de la Reproduction et des Comportements, Université de Tours, Nouzilly, France; 2 Université de Paris, Institut Cochin, INSERM, CNRS, Paris, France; 3 Institut de Recherches Servier, Croissy sur Seine, France; Duke University School of Medicine, UNITED STATES

## Abstract

Melatonin (MLT) is a biological modulator of circadian and seasonal rhythms and reproduction. The photoperiodic information is detected by retinal photoreceptors and transmitted through nerve transmissions to the pineal gland, where MLT is synthesized and secreted at night into the blood. MLT interacts with two G protein-coupled receptors, MT_1_ and MT_2_. The aim of our work was to provide evidence for the presence of MLT receptors in the ovine pineal gland and define their involvement on melatonin secretion. For the first time, we identified the expression of MLT receptors with the specific 2-[^125^I]-MLT agonistic radioligand in ovin pinealocytes. The values of Kd and Bmax are 2.24 ± 1.1 nM and 20 ± 6.8 fmol/mg. MLT receptors are functional and inhibit cAMP production and activate ERK1/2 through pertussis toxin-sensitive G_i/o_ proteins. The MLT receptor antagonist/ inverse agonist luzindole increased cAMP production (189 ± 30%) and MLT secretion (866 ± 13%). The effect of luzindole on MLT secretion was additive with the effect of well-described activators of this pathway such as the β-adrenergic agonist isoproterenol and the α-adrenergic agonist phenylephrine. Co-incubation of all three compounds increased MLT secretion by 1236 ± 199%. These results suggest that MLT receptors are involved in the negative regulation of the synthesis of its own ligand in pinealocytes. While adrenergic receptors promote MLT secretion, MLT receptors mitigate this effect to limit the quantity of MLT secreted by the pineal gland.

## Introduction

Melatonin (MLT) is a biological modulator of circadian and seasonal rhythms, sleep, reproduction, mood and has antioxidant activity [1–8]. The photoperiodic information is detected by retinal photoreceptors and transmitted through nerve transmissions to the pineal gland, where MLT is synthesized and secreted at night into the blood. The synthesis of melatonin is regulated by α1- and β1-adrenoreceptors which induce the phosphorylation of Aralkylamine N-Acetyltransferase (AANAT) through cyclic AMP-dependent protein kinase (PKA), a key enzyme in the synthesis of MLT. The pineal gland is the major site of MLT synthesis in the body, but other tissues, such as the retina, salivary glands, platelets, lymphocytes and gastrointestinal tract, also synthesize this hormone locally [9–11].

MLT exerts its physiological effects through two G protein-coupled-receptors, MT_1_ and MT_2_ [12, 13], that bind MLT with a high affinity in the pM-nM range, depending on several factors such as the tissues, cell lines studied and the number of receptors expressed [14–16]. In addition, a cytoplasmic enzyme named MT_3_ or QR2, involved in the detoxification of quinones by reduction, also binds MLT, albeit with a lower affinity than MT_1_ and MT_2_ receptors [17, 18].

MT_1_ and MT_2_ receptors are mainly coupled with Pertussis toxin -sensitive, Gi/o proteins. They can also couple to other G proteins such as Gq and to soluble guanylate cyclases [19–21].

MT_1_ and MT_2_ receptors have been detected in many different tissues and in various species such as sheep, a well-adapted species for in vivo studies about MLT action. Numerous data on MT receptors are focused mainly on the control of seasonal reproductive function, particularly through receptors located in the hypothalamus, and the pars tuberalis [22–32]. More unexpected, RT-PCR studies demonstrated the presence of mRNA encoding MT_1_ and MT_2_ receptors in the pineal gland, the site of melatonin synthesis in sheep [33]. This suggests a role of melatonin on its own synthesis. However, no data reporting the presence of the MT receptors at the protein level and their functional characteristics in the pineal gland are available unlike other species such as human [34].

Hence, the aim of our work was to provide evidence for the presence of MT receptors in the ovine pineal gland and define their functional and pharmacological properties. Moreover, the involvement of MT receptors on melatonin secretion was investigated.

## Materials & methods

### Reagents

Antibiotic-antimycotic solution, trypsin-EDTA, Dulbecco’s Modified Eagle Medium/Ham F12 without phenol red (DMEM /F12), Hank’s balanced salt solution (HBSS), bovine serum albumin (BSA), fetal bovine serum (FBS) and protease inhibitor cocktail were obtained from Gibco-Life Technologies (France). Collagenase A was purchased from Roche Diagnostics (Meylan, France). MLT, forskolin, isoproterenol, propranolol, phenylephrine, prazosin, p-chlorophenyl alanine (PCPA), reserpine, 3-Isobutyl-1-methylxanthine (IBMX), Triton-X100, Tween 20, Saponin, Igepal, poly-L-ornithine, DNAse I, L-ascorbic acid and perchloric acid were obtained from Sigma-Aldrich (Saint Quentin Fallavier, France), and 4P-PDOT and luzindole from Tocris (Bristol, UK). Hybond-C Extra nitrocellulose and enhanced chemiluminescence (ECL) detection reagents were purchased from GE Healthcare Life Sciences (Vélizy-Villacoublay, France) and BCA reagents for protein determination from Interchim (Montluçon, France). Radioimmunoassay kit for determining cAMP concentrations was provided by Immunotech s.r.o (Praha, Czech Republic). The two radioligands 2-[^125I^]-MLT (specific activity: 2,200 Ci.mmol^-1^) and [^125I^]-S70254 (specific activity: 2,175 Ci.mmol^-1^) were purchased from PerkinElmer (Courtaboeuf, France) and ANAWA Trading SA (Wangen/Zürich, Switzerland) respectively. S70254 was given by Institut de Recherche Servier (IDRS) (Croissy sur Seine, France).

### Antibodies

Primary antibodies raised against pERK1/2 and ERK 1 were obtained from Cell Signaling Technology (Leiden, Netherlands) and Santa Cruz Biotechnology (Heidelberg, Germany) respectively. Rabbit anti-tryptophane-5-hydroxylase antibodies (TPH) were obtained from Santa Cruz Biotechnology (Heidelberg, Germany). Rabbit and mouse anti-glial fibrillary acidic protein antibodies (GFAP) were from Dako and Sigma. Anti-rabbit and anti-mouse biotinylated secondary antibodies were obtained from Vector laboratories (Nanterre, France). The streptavidin-peroxidase conjugate was purchased to Alpha Diagnostic International (Texas, USA) and Santa Cruz Biotechnology (Heidelberg, Germany). DyLight 488 Streptavidin was from Vector laboratories (Nanterre, France). Texas Red anti-rabbit secondary antibodies were obtained from Jackson Immunoresearch Laboratories (Pennsylvania, USA).

### Primary culture of ovine pineal gland cells

The pineal glands of lambs aged 3 to 6 months, from Ile-de-France or Solognote breeds and mixed sexes (90% males) were obtained from a local slaughterhouse (Perche Vendômois) throughout the year. Glands were collected at around 5:00 a.m. They were immediately placed into ice-cold DMEM/F12 without phenol red. After several washes, pineal glands were sliced into small pieces which were then placed in DMEM/F12 supplemented with antibiotic-antimycotic solution (100 IU/ml penicillin, 100 μg/ml streptomycin and 0.25 μg/ml fungizone), 0.004% DNase I, 0.1% trypsin-EDTA and 0.1% collagenase A for 1 h at 37°C. To help the enzymatic digestion, mechanical disruption of tissue was done every 20 min with hypodermic needles. The cells were then filtered with a sterile gauze, centrifuged at 250 x *g* for 5 min at 4°C, washed and resuspended in DMEM/F12 supplemented with antibiotic-antimycotic solution and 10% FBS. To remove astrocytes from the primary cultures, the dissociated cells were deposited in a Petri dish for 1–2 hours at 37°C. Pinealocytes remained in suspension while astrocytes attached to the culture flasks. Then, pinealocytes were seeded in culture wells coated with 0.01% poly-L-ornithine and cultured for 8 to 12 days in the same medium at 37°C with 95% O_2_ / 5% CO_2_ before starting the assays.

### Immunocytochemistry

After washing, 2x10^5^ cells/well were fixed with 4% Paraformaldehyde in PBS. After washes in PBS, inhibition of endogenous peroxidases was performed with 6% H_2_O_2_. Cells were then incubated in blocking solution (normal sheep serum diluted 1:15 in PBS) containing 0.2% of saponin and 2% BSA for 1 hour with gentle shaking. Immunostaining was carried out by incubating cells with 1:600 rabbit anti-tryptophane-5-hydroxylase (TPH) or anti-glial fibrillary acidic protein (GFAP) in PBS buffer containing 0.2% of saponin and 0.5% BSA overnight at 4°C. After washing, cells were incubated with 1:1000 anti-rabbit biotinylated secondary antibody in PBS buffer supplemented with 0.2% of saponin and 0.5% BSA for 90 min at room temperature, followed by incubation with peroxidase conjugated streptavidin for 60 min at room temperature. Cells were rinsed 3 times for 5 minutes and incubated for 5 min with a 0.05 M Tris HCl solution pH 7.8 containing 0.02% 3,3′-diaminobenzidine tetrahydrochloride (DAB) and 0.015% hydrogen peroxide (H_2_O_2_). After washing, the preparations were observed by white-light optical microscope. For controls, non-immune normal rabbit serum (d: 1/600) was substituted to the primary antibodies.

Immunofluorescence labelling was carried out as described above with slight modifications, mainly regarding antibodies and detection systems. Briefly, fixed and permeabilized cells were incubated with 1:600 rabbit anti-tryptophane-5-hydroxylase (TPH) or mouse anti-GFAP in PBS buffer containing 0.2% saponin and 0.5% BSA. To detect anti-TPH antibodies and anti-GFAP antibodies, cells were incubated with 1:200 texas red conjugated anti-rabbit antibody or with 1:1000 biotinylated anti-mouse antibody, respectively, for 90 min at room temperature. This was followed by incubation with 1:300 DyLight 488 streptavidin for 60 min at room temperature. Cells were then mounted on slide with Vectashield mounting medium containing DAPI (Sigma-Aldrich, Saint Quentin Fallavier, France). As controls, the primary or the secondary antibodies were omitted. Immunolabelling was observed with confocal microscopy (LSM700, Zeiss) with DAPI, DyLight 488 and texas red filter sets. The images were processed with the ZEN software (version 2010).

### Radioligand binding assays

For radioligand assay, 8x10^5^cells were washed with HBSS and incubated with binding buffer (50 mM Tris-HCl pH 7.4, 5 mM MgCl_2_, 1 mM EDTA) for 1h at 37°C. Saturation binding assays were performed with 2-[^125I^]-MLT alone or with an excess of unlabelled MLT to evaluate non-specific binding. Similar experiments were done with the MT_2_ receptor agonist [^125^I]-S70254 ± S70254. Briefly, 0.08–1.5 nM radioligands alone or with 10 μM unlabelled ligand were incubated in binding buffer for 1 h at 37°C. The reaction was stopped by aspiration of the medium. Cells were incubated in a cold-acid buffer (50 mM glycine, 150 mM NaCl) for 3 min at 4°C and supernatants were stored to quantify the number of radioligands bound on cell surface. Then, cells were lysed in 1 M NaOH for 2 h at 37°C to measure intracellular ligand-receptor complexes. Radioactivity was measured with a Wizard gamma counter (Perkin Elmer) Data were analysed by using the PRISM software (GraphPad Software Inc., San Diego, CA, USA) and results were presented as the mean of duplicate measurements. The density of binding sites (B_max_) and the dissociation constant of the radioligand (K_D_) values were calculated according to the method of Scatchard (F test in GraphPad Prism, version 5).

### Depletion of pineal MLT in cell culture

The functional characterization of MT receptors in ovine primary pinealocytes cultures required the suppression of the pineal MLT secreted into the culture medium. Before each experiment, cells were washed several times. In details, 24 h before the experiment, the culture medium was replaced by DMEM/F12 without phenol red. Cells were washed 3 times with HBSS at 37°C and incubated with Tris buffer for 1 h at 37°C. The experiments on the functional activity of the MT receptors were then performed.

### cAMP assay

For the measurement of intracellular cAMP accumulation, 1x10^5^ cells were washed several times with HBSS and incubated in DMEM/F12 supplemented with 100 μM IBMX, 100 mg/ml L-ascorbic acid and different compounds for 1 h at 37°C in a 5% CO_2_ atmosphere. The pharmacological agents used were 10^−7^ M MLT, 1 μM forskolin, 10^−7^ M MLT plus 1 μM forskolin or 1 μM luzindole. To assay MTR coupling to G_i/o_, cells were pre-incubated with 5 ng/ml PTX for 3 h at 37°C and then incubated with 1 μM forskolin plus 10^-7^M MLT for 1 h at 37°C. After washes with HBSS, cells were incubated with 50 μl of cold perchloric acid 2N for 30 min at room temperature. The pH was then neutralized with 50 μl KOH 1N. Culture dishes were centrifuged at 3,000 × *g* for 10 min at 4°C. Supernatants were collected and centrifuged at 10,000 × *g* for 7 min at 4°C. cAMP accumulation in the supernatant was then measured with a radioimmunoassay kit.

### Phospho- ERK1/2 measurements

Cells (4x10^5^ per well) were starved in serum free DMEM/F12 overnight, then stimulated at 37°C with 10^−7^ M MLT for 5 min and lysed overnight at 4°C in lysis buffer containing 150 mM NaCl, 10 mM Tris pH 7.4, 1 mM EDTA, 1mM EGTA, 1% Triton X-100, 0.5% Igepal, 100 mM sodium fluoride, 10 mM sodium pyrophosphate, 10 mM sodium orthovanadate and proteases inhibitors cocktail. To assay the involvement of protein Gi, starving cells were pre-incubated with 5 ng/ml PTX for 3 h at 37°C and then incubated with 10^-7^M MLT for 5 min at 37°C and lysed overnight as described above. For each sample, protein concentration was determined by BC Assay.

### Western blot analysis

Ten μg proteins denatured by heating at 100°C for 5 min were resolved by 10% SDS/PAGE and transferred to a Hybond-C nitrocellulose membrane. Membranes were blocked with 5% non-fat dry milk diluted in PBS supplemented with 0.1% Tween 20 and incubated with anti-pERK1/2 (1:3,000) or anti-ERK 1 (1:2,000) overnight at 4°C. After washes, membranes were incubated with either anti-rabbit horseradish peroxidase-conjugated (1:5,000) or biotinylated anti-mouse antibody (1:250) plus peroxidase-conjugated streptavidin (1:10,000) for 1 h at room temperature. Immunoreactivity was revealed using an enhanced chemiluminescence reaction ECL. Intensity of the bands was quantified by densitometry using the TotalLab Quantsoftware (TotaLlab, Version 12.2, Newcastle-upon-Tyne, UK).

### Measurement of MLT in the culture medium

Cells were seeded at a density of 2x10^5^ cells/well. Before the stimulation, the cells were washed several times with HBSS to suppress extracellular MLT. Then, the cells were incubated for 1 h at 37°C with HBSS supplemented with 50 μM L-ascorbic acid and various compounds. The pharmacological agents used were forskolin (adenylyl cyclase catalytic activator, 1 μM), isoproterenol (β-adrenergic receptor agonist, 1 μM), propranolol (β-adrenergic receptor antagonist, 1 μM), phenylephrine (α1-adrenergic receptor agonist, 1 μM), Prazosin (α1- adrenergic receptor antagonist, 1 μM) and luzindole (MLT receptor antagonist, 1 μM). All the supernatants were collected and stored at -80°C.

The MLT concentration was measured in the supernatants by a direct radioimmunoassay (RIA) using 2-[^125I^]-MLT as reported previously [35–37]. Briefly, 100 μl of culture supernatants were incubated with 300 μl of 2-[^125I^]-MLT (20,000 c.p.m/tube, 21 pM, specific activity: 2,200 Ci.mmol^-1^) and 100 μl of rabbit polyclonal anti-MLT diluted at 1:400,000 [37]. After 18 h of incubation at 4°C, 1 ml of sheep anti-rabbit whole serum diluted at 1:300 was added for 1 h at 4°C. All reactions were stopped by centrifugation at 2,800 x *g* for 30 min at 4°C and radioactivity was measured using a gamma counter. The sensitivity of the assay was 4 pg/ml and the mean intra- assay coefficient of variation was 11.3%.

### Statistical analyses

All results were analyzed by using the PRISM version 5 software (GraphPad Software Inc., San Diego, USA). Data were expressed as means ± SEM. Statistical analysis was performed with Kruskal-Wallis tests followed by the Dunn’s multiple comparison tests with statistical significance set at P<0.05. *P<0.05, **P<0.01, ***P<0.001.

## Results

### Cytological characterization of primary culture of lamb pineal gland cells

**Fig 1:**
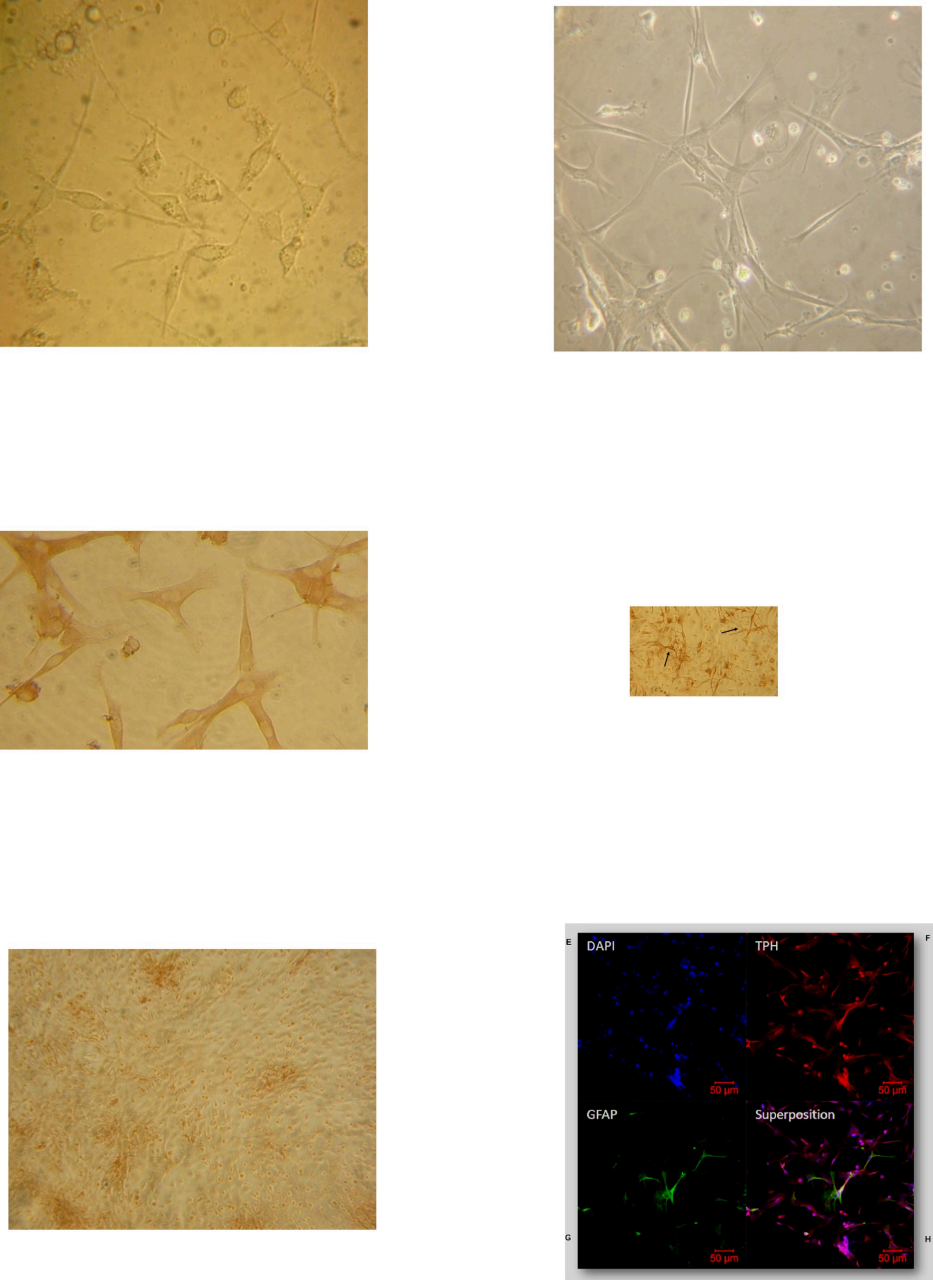
Cytological characterization of primary culture of ovine pineal gland cells. Five (A) and eight days (B) primary culture of ovine pineal glands were observed by optical microscopy. Cells from ovine pineal glands were immunolabelled with anti-tryptophan hydroxylase (TPH, C) or anti- glial fibrillary acidic protein (GFAP, D) antibodies and detected by a peroxydase-coupled secondary antibody. Non immun serum (NIS) was used as a negative control (Fig 1E). Magnification was x400 in A and C, x200 in B and x100 in D. Pinealocyte were labelled with an anti-TPH antibody (G) detected by a cyanin-coupled secondary antibody, while astrocytes were labelled with an anti-GFAP antibody (H) detected by fluorescein-coupled secondary antibody. Nuclei were stained with DAPI (F). The merge of the three labelings is shown in I. Maginifcation is x10. Scale bar 50 μm. Astrocytes (arrow).

Consistent with the literature, the cells in our primary culture were fusiform with a large central nucleus (Fig 1A). After enzymatic dissociation of pineal glands, pinealocytes began to spread on their support from 4–5 days of culture and were perfectly elongated after 8 days (Fig 1B). Cells were characterized by immunocytochemistry using specific markers of pinealocytes (tryptophan hydroxylase, TPH) (Fig 1C) and of astrocytes (glial fibrillary acidic protein, GFAP) (Fig 1D). Immunocytochemistry (Fig 1C–1E) and immunofluorescence (Fig 1F–1I) show that the cell cultures were mainly composed of pinealocytes (85–90%). The remaining cells were glial cells such as astrocytes, as revealed by their starry shape and their long and slender processes. In some instances, pinealocytes were wrapped and intertwined with astrocytes, as shown by the merged fluorescence (Fig 1I). Conjunctive tissue was also present in our cultures. To avoid overwhelming growth of fibroblasts, that has been shown to occur after 15 days of culture [38, 39], we chose to explore the MT receptor functionality in cells maintained in culture for 8 to 12 days.

### Binding properties of MT_1_ and MT_2_ in pinealocytes

To explore MT receptor functionality in a controlled manner in primary ovine pinealocytes, we first had to deplete cell media of MLT, since it is constitutively secreted into the medium. Therefore, cells were washed several times with buffer prior to functional assays. RIA assays showed that MLT concentration equal to 4 pg/ml (1.7 x 10^-11^M) were still present in the medium after extensive washes.

**Fig 2:**
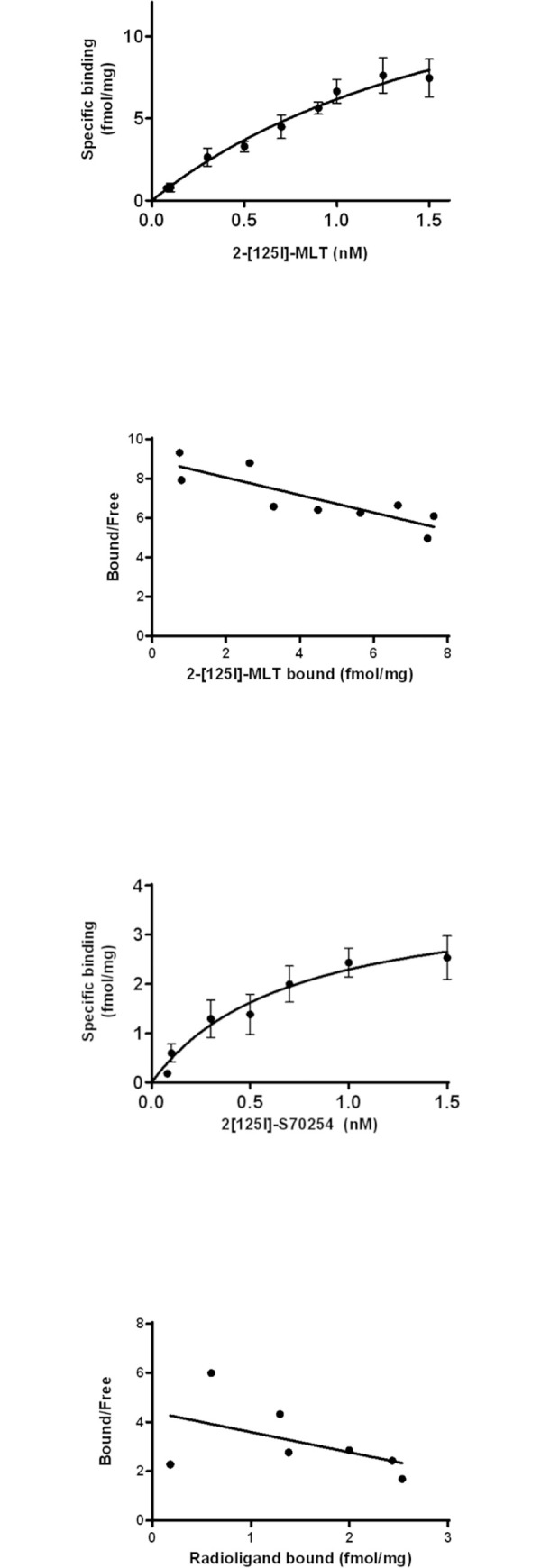
Binding properties of MT_1_ and MT_2_ in ovine pinealocytes. Saturation curves performed with 2-[^125I^]-MLT (A) and [^125I^]-S70254 (C) on primary culture of ovine pineal gland cells at 37°C for 1 h. Specific binding is represented. Scatchard plot is associated with the saturation curves (B and D). The experiments are performed in duplicates and are repeated five times.

MLT binding sites were identified in pinealocytes by using two radioligands, the classical, non-selective MT receptor agonist 2-[^125^I]-MLT [40, 41], and the specific human MT2 agonist [^125^I]-S70254 [16, 42]. The 2-[^125I^]-MLT, known to exhibit a high affinity for both MT_1_ and MT_2_ receptors showed a monophasic saturation curve, a Scatchard linear regression with a Kd value of 2.24 ± 1.1 nM and a Bmax of 20 ± 6.8 fmol/mg (Fig 2A and 2B). Similar results were obtained when cells were pre-treated with the PCPA tryptophan hydroxylase inhibitor and reserpine a blocker of the vesicular monoamine transporter, in order to block the melatonin synthesis (S1 Fig), suggesting that endogenous MLT production does not influence the binding characteristics of MT receptors for 2-[^125I^]-MLT.

The saturation curve obtained with [^125I^]-S70254 suggested that MT2 receptors are expressed in pinealocytes (Fig 2C). Even though the dispersion of the values around the regression curve did not allow to define reliable Kd and Bmax for MT2 receptors, the amount of MT2 receptors expressed appeared to be lower than the one measured with 2-[^125I^]-MLT (Fig 2D). Together, these data suggest that both MT_1_ and MT_2_ receptors are expressed in our primary culture of ovine pinealocytes.

### Pharmacological characterization of MT receptors in pinealocytes

**Fig 3:**
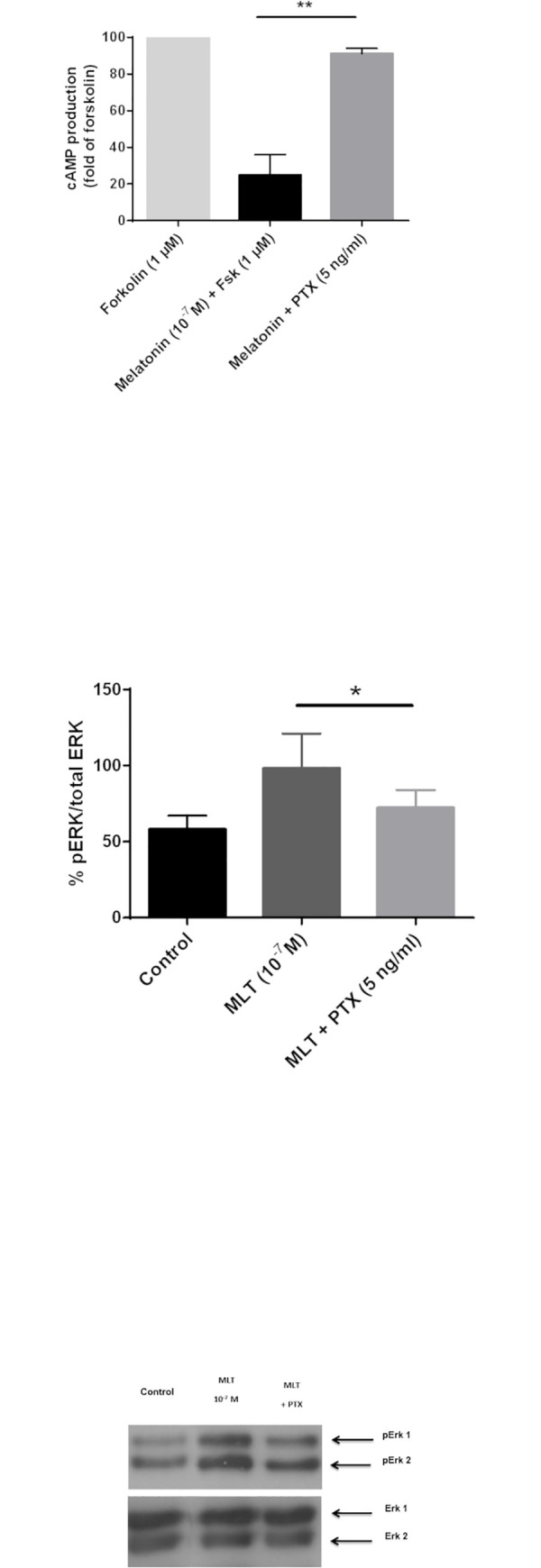
Coupling of MT receptor to Gi proteins in pinealocytes. (A) Effect of 10^−7^ M MLT on cAMP production induced by 1 μM of Forskolin (Fsk). Effect of pre-treatment with Pertussis toxin (PTX; 5 ng/ml; 3 h at 37°C) on cAMP accumulation following stimulation with 10^−7^ M MLT and 1 μM of Fsk (1 h at 37°C). Each histogram is presented as a percentage of the maximal response to 1 μM of forskolin (100%). B) Effect of 10^−7^ M MLT or of a pre-treatment with Pertussis toxin (PTX; 5 ng/ml; 3 h at 37°C) and then 10^−7^ M MLT (5 min) on the level of phosphorylated ErK 1/2. (C) The Western blot is a representative experiment. Columns and bars represent mean ± s.e.m. values of data obtained from duplicate determinations of cAMP in 5 different experiments (A) and 3 assays for the phospho-Erk1/2 level (B and C). *P<0.05 in Kruskal-Wallis tests followed by the Dunn’s multiple comparison tests.

We next evaluated the impact of MLT on the forskolin-induced cAMP production in pinealocytes. The ability of cells to produce intracellular cAMP *via* direct activation of adenylate cyclase was assessed with 1 μM forskolin. The cAMP concentration was 2.5-fold higher than the one measured in control condition (32 ± 6.2 nM with Fsk *vs* 13 ± 2 nM). MLT inhibited approximately 70–90% of the forskolin-induced cAMP production (Fig 3A), which supports G_i/o_ coupling of MTRs, as previously reported in other cell types [20, 43–46]. Consistently, the pre-incubation of cells with 5 ng/ml of pertussis toxin (PTX) to uncouple G_i/o_ proteins fully blocked the ability of MLT (10^−7^ M) to inhibit forskolin–stimulated cAMP production (91 ± 3% with PTX *vs* 25 ± 11% with Fsk + MLT) (Fig 3A). Similarly, the activation of ERK1/2 MAP kinases by MLT was transduced, as least in part, through G_i/o_ coupling of ovine MT receptors, since ERK phosphorylation was partially inhibited (72 ± 12% with PTX *vs* 98 ± 9% with MLT) by preincubation of pinealocytes with 5 ng/ml PTX (Fig 3B and 3C and S2A and S2B Fig). Altogether, these results show that the MT receptors expressed in the lamb primary pinealocyte cultures are coupled with a pertussis toxin-sensitive G protein (G_i/o_) and induce the activation of the ERK1/2 pathway *via* the stimulation of G_i/o_ proteins.

**Fig 4:**
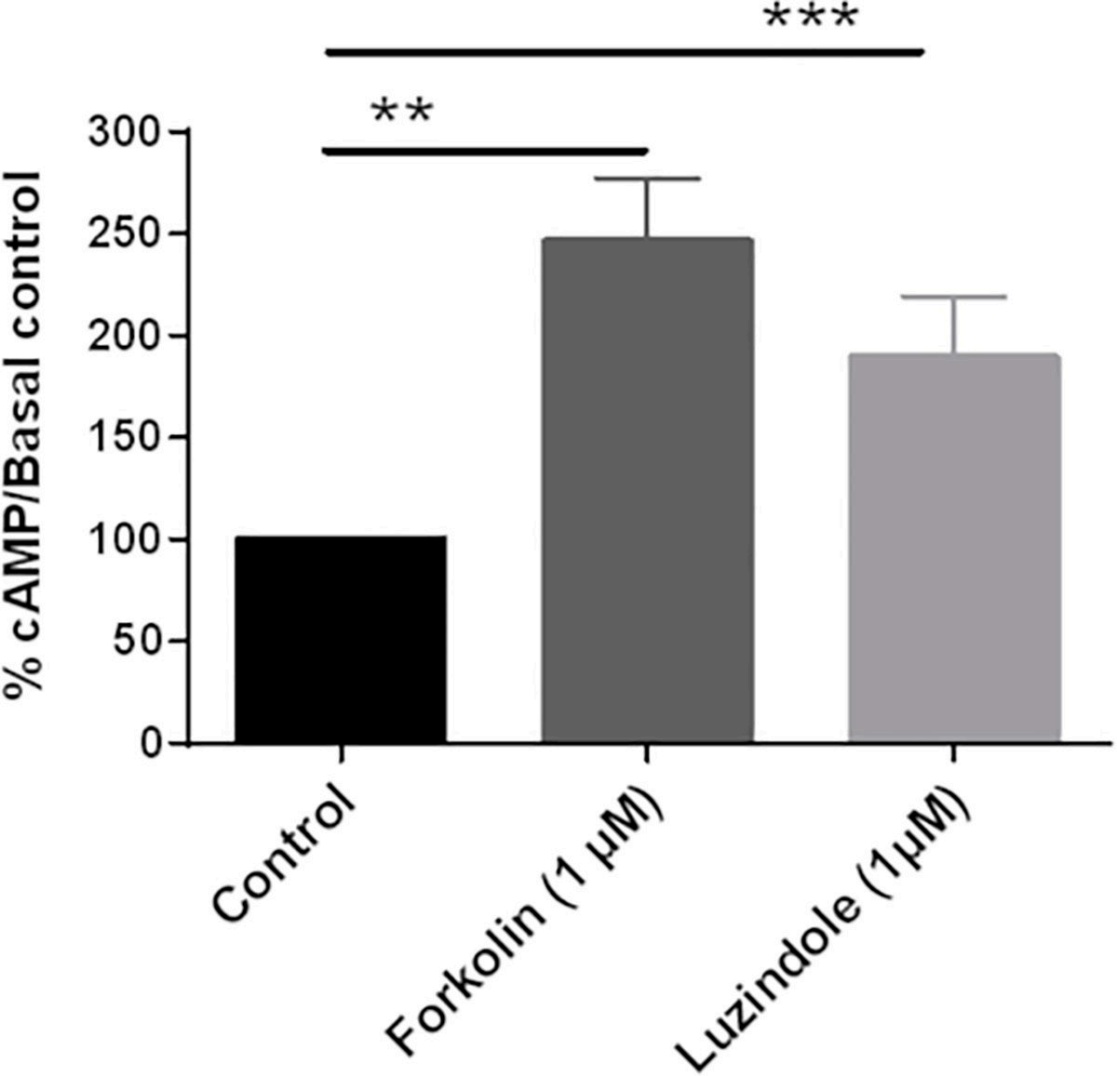
Inhibition of cAMP production mainly mediated by MT receptors. Effect of 1 μM Forskolin or 1 μM luzindole on basal cAMP production. Each histogram is presented as a percentage of the basal cAMP accumulation (control; 100%). Columns and bars represent mean ± s.e.m. values of data obtained from duplicate determinations of cAMP in 5 different experiments. *P<0.05 in Kruskal-Wallis tests followed by the Dunn’s multiple comparison tests.

The functional expression of MT receptors in the ovine pinealocytes were determined using the non-selective MT_1/2_ receptor antagonist, luzindole [19, 47–49] (Fig 4). Incubation of the cells with 1 μM of luzindole induced a significant increase in the cAMP production (189 ± 30%). This result confirms the expression of active receptors in ovin pinealocytes.

### Secretion of MLT

**Fig 5:**
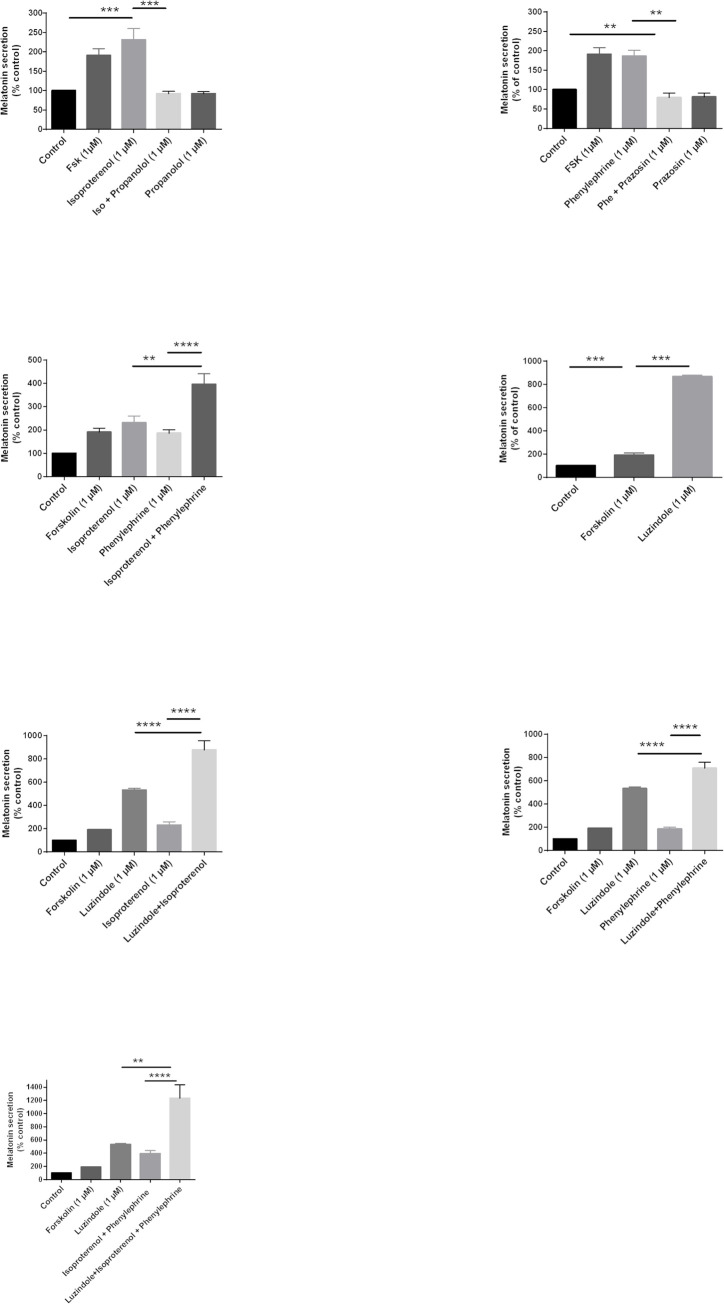
Regulation of MLT synthesis. Effects of adrenergic and/or MT receptors on MLT synthesis. Receptor activity was stimulated with isoproterenol for β- adrenergic receptors (A), phenylephrine for α1-adrenergic receptors (B), both isoproterenol and phenylephrine (C), luzindole for MT_1/2_, both luzindol and isoproterenol for MT and β- adrenergic receptors (E), both luzindol and phenylephrine for MT and α1-adrenergic receptors (F), and both luzindol, isoproterenol and phenylephrine for MT and β- and α1-adrenergic receptors (G). Each histogram is presented as a percentage of the control MLT secretion (Control, 100%). Columns and bars represent mean ± s.e.m. values of data obtained from duplicate determinations of cAMP in 5 different experiments. *P<0.05 in Kruskal-Wallis tests followed by the Dunn’s multiple comparison tests.

The synthesis of MLT is well known to be regulated by α1- and β1-adrenergic receptors in the pineal gland of different species [50–52]. We assessed whether this regulatory system was conserved and functional in primary cultures of ovine pinealocytes. First, cells were incubated with isoproterenol (1 μM), a specific agonist of β-adrenergic receptors. Isoproterenol significantly increased the level of secreted MLT compared to the basal state (231 ± 29%), and this effect was completely abolished by the specific β-adrenergic receptor antagonist propanolol (92 ± 6%) (Fig 5A).

Similarly, the α1-adrenergic receptor agonist phenylephrine enhanced MLT secretion (186 ± 15%) and this effect was abolished by the co-incubation with prazosin (79 ± 12%), a specific α1-adrenergic receptor antagonist (Fig 5B). As expected co-incubation of pinealocytes with both isoproterenol and phenylephrine led to an additive effect on MLT secretion compared to each agonist alone (396 ± 46% vs 231 ± 29% with isoproterenol or 186 ± 15% with phenylephrine) (Fig 5C).

These data show that both the α1- and β-adrenergic receptors regulate the synthesis of MLT in primary cultures of pinealocytes, as described in the literature for pineal glands of mammals [51, 53, 54] and can work in an additive manner. We investigated whether MT receptors can themselves regulate the synthesis and secretion of MLT in pinealocytes. The use of the MT receptor antagonists showed that luzindole strongly increased the level of MLT secretion (866 ± 13%) (Fig 5D). These results suggest that the MT receptors are responsible for the inhibition of MT synthesis in primary pinealocytes and that the residual MLT present in the culture after extensive washes and/or induced by adrenergic agonists is exerting a potent brake on MLT secretion by pinealocytes.

To determine whether adrenergic and MLT receptors might exert a coordinated action on MLT synthesis, cells were co-incubated with luzindole and either isoproterenol or phenylephrine. Simultaneous β-adrenergic stimulation and luzindole treatment increase the level of secreted MLT to 879 ± 78% compared to luzindole 533 ± 16% alone or isoproterenol alone 231 ± 29% (Fig 5E). Similar observations were made with the co-addition of the α-adrenergic agonist phenylephrine and luzindole, reaching the MLT secretion to 708± 51% compared to phenylephrine alone 186 ± 16% or 533 ± 16% with luzindole only (Fig 5F). Interestingly, when pinealocytes were incubated simultaneously with the 2 adrenergic receptors agonists with luzindole, reaching 1236 ± 199% of MLT produced against 396 ± 45% with isoproterenol and phenylephrine (Fig 5G).

These results suggest that adrenergic receptors on the one hand and MT receptors on the other hand exert opposite regulations on MLT secretion.

## Discussion

Our study identifies for the first time active MT receptors in ovine pinealocytes. Interestingly, these results highlight a new role for the MT receptors in the pineal gland where they negatively regulate their own ligand synthesis. While stimulation of α and β-adrenergic receptors elevate intracellular cAMP via a Gs-dependent mechanism to favor MLT secretion, MT receptor activity seems to mitigate this effect to limit the quantity of MLT secreted by the pineal gland.

MLT-stimulated MT receptors are coupled to PTX-sensitive G_i/o_ proteins, which results in decreased intracellular cAMP production and increased Erk1/2 phosphorylation. This type of coupling is classically described for this family of receptors in all species and in cell lines [21, 43, 46, 55, 56] and native tissues such as pars tuberalis and retina [46, 57].

The two radioligands that were used in this study, 2-[^125I^]-MLT or [^125I^]-S70254, bind to a single high affinity binding site. In the pineal gland, the affinity of MT receptor binding to 2-[^125I^]-MLT is in the nanomolar range, and is comparable to the binding affinity described in rabbit retina [14, 47], another tissue that synthesizes MLT. A picomolar affinity has been described in cell lines expressing recombinant MT receptors [15, 16, 33, 58], as well as in pars tuberalis, a native tissues that do not synthesize MLT [24, 26, 59]. We were not able to identify such a high affinity site in pinealocytes. In our study, [^125I^]-S70254 did not allow to define a binding affinity in pinealocyte cells in contrast to 2-[^125I^]-MLT. Legros and collaborators encountered the same difficulties in sheep retina cells although this tissue expresses more MT receptors than pinealocytes. This radioligand dissociates rapidly and completely from hMT_2_ receptors at 37°C unlike 2-[^125I^]-MLT which partially dissociates [16, 20, 42]. Furthermore, the affinity of this radioligand for ovine MT_2_ receptors is in the nanomolar range (21.3 nM). All these characteristics do not seem suitable for obtaining a stable saturation plateau especially when receptors are weakly expressed. In these conditions, a reproductible and reliable Scatchard analysis is difficult to obtain.

Our results show that MLT binding sites are expressed at a low density in pineal gland. Unfortunately, we were unable to determine the precise amount of MT_2_ receptors expressed in the pineal gland. Cogé and collaborators were the first to detect the presence of the MT_2_ mRNA by RT-PCR in the ovine pineal gland and found that MT_2_ is much less abundant than MT_1_ [33]. Given the difficulties of several labs to detect MT_2_ expression in the pineal gland, it is reasonable to assume that MT_1_ is also predominant receptor on the protein level. MT receptors are 4 times less expressed in pinealocytes than in ovine pars tuberalis [60]. Noteworthy, the number of receptors is underestimated because the radioligands used are agonists. They bind only to the active forms of the receptors. A reliable antagonist would be a necessary tool to define the total number of receptors expressed in the pinealocytes. A difference of 4 times in the amount of receptors expressed does not necessarily translate in 4 times lower functional response. Often maximal effects are already reached at low receptor expression levels due to the amplification of the initial signal on the level of the signaling pathway. In addition, the maximal response depends on the cellular context, a phenomenon described as system bias [21]. It is therefore very difficult to compare the maximal effect of MT receptors between pinealocytes and the pars tuberalis. It is well established in different cellular models that MLT inhibits the production of cAMP *via* G_i/o_-protein-coupled MT receptors and conversely induces an increase in the level of phosphorylated Erk1/2 [21, 44, 46]. We observed the same effects with pineal gland cells. The decrease of cAMP production is inhibited by luzindole indicating the involvement of MT_1/2_ receptors in the MLT-related response. 4-PPDOT is a MT_2_-selective antagonist, in particular for human receptors. However, in sheep, 4-PPDOT has only a 10-fold higher affinity for oMT_2_ receptors than for oMT_1_ receptors and shows even the same efficacy on both receptors [33, 60]. 4-PPDOT is therefore unable to discriminate between the two ovine receptors. Our data as well as those published for 4-PPDOT, unfortunately do not allow us to conclude on the relative contribution of oMT_1_ vs oMT_2_ in the feedback regulation of MLT secretion in sheep pinealocytes.

Our data support that primary pinealocytes cells in culture maintained their functional properties and endogenous regulatory processes. Indeed, they are able to synthesize and secrete MLT and to regulate this synthesis upon the activation of the α1- and β-adrenergic receptors. We also show that MLT secretion is higher when both α1- and β-adrenergic receptors are activated. We did not observe any seasonal variability in the capacity of pinealocyte cultures to produce melatonin upon activation of these receptors. Moreover, concomitant activation of both adrenergic receptors and inhibition of MT receptors clearly resulted in a strongly enhanced MLT secretion, confirming an unexpected negative regulatory mechanism of the MT receptors in MLT secretion. Melatonin synthesis is limited by the activity of 3 main enzymes: TPH (tryptophan hydroxylase) [61], AA-NAT (arylalkylamine N-acetyltransferase) [51] and HIOMT (hydroxyindole-O-methyltransferase) [62, 63]. The enzymatic activity of these enzymes is regulated by adrenergic receptors [61, 64, 65] with cAMP/PKA. Previous studies have shown in non-rodent mammalian species that phosphorylation of AANAT results in its association with 14-3-3 protein and in the enhancement of MLT synthesis [66]. Ganguly and collaborators showed that there are large dynamic pools of AANAT in phosphorylated and unphosphorylated forms in ovine pinealocytes [67]. Thus, the co-stimulation of cells by adrenergic receptor agonists and luzindole could increase the number of phosphorylated AANAT, the formation of AANAT/14-3-3 protein complexes and the MLT synthesis. On another hand, the protein kinase C (PKC), was also described for the regulation of the activity of AANAT in bovine pinealocytes [68]. It would be interesting to determine whether PKC and MAP kinases are also involved in this regulation in ovine cells. The 3 main enzymes are also regulated by adrenergic receptors with store operated channels (SOCs) [69], calcium influx through high voltage-activated calcium channels [70], 5-HT secretion and 5-HT2 receptor activation [71]. The stronger synthesis of MLT in the presence of luzindole suggests that MT receptors could regulate the same intracellular actors as adrenergic receptors but they would modulate them differently. The increase of melatonin secretion could also be due to the regulation of other actors. Yamada and colleagues showed that metabotropic glutamate 3 receptors inhibit MLT synthesis by inhibiting the cAMP cascade induced by adrenergic receptors [72, 73]. These receptors are activated by glutamate which is secreted from ovine pinealocytes following the activation of nicotinic receptors. MT receptors could therefore inhibit MLT synthesis: 1) by regulating the functional activity of nicotinic receptors; 2) by increasing glutamate secretion from pinealocytes; 3) by activating metabotropic glutamate receptors 3 directly and inhibit MLT synthesis. So far, the negative regulatory mechanism of the MT receptors in MLT secretion remains elusive and further analyses are required to identify the regulatory processes involved.

The culture we developed for the lamb pineal gland is composed of several cell types including pinealocytes and interstitial cells as described in the literature for several species [74, 75]. A single cell transcriptome analysis of rat pineal has showed that 2 types of pinealocytes, called αand β, could be differentiated by the expression of the *Asmt* gene, which encodes another major enzyme in MLT synthesis, Acetylserotonin O-Methyltransferase (ASMT) [75]. The elevated *Asmt* expression in α-pinealocytes suggests that these cells have an enhanced capacity to catalyze the formation of MLT from N-acetylserotonin compared to β-pinealocytes [76]. It would be interesting to assess whether or not these two populations can be observed in our culture system and, if yes, to study the expression of MT receptors in each of the pinealocyte populations and the regulation of MLT synthesis in these populations under different physiological conditions (day-night, seasonality).

Three types of astrocytes, designated as α, β, and γ, were also identified in the transcriptomic study [75]. In our ovine primary cultures, the astrocytes seem to coat the pinealocytes suggesting a regulatory, protective or supporting role for these cells as described in the literature for neurons [77–80]. Astrocytes play a role in the modulation of MLT synthesis *via* catecholamines and glutamate [80, 81]. These effects are important in sheep and human pineal glands but not in rodents. The effects of glutamate involve paracrine interactions between pinealocytes and astrocytes through TNF-α release [82]. With that in mind, one can hypothesize that astrocytes could regulate extracellular MLT levels around the pinealocytes by trapping and /or metabolizing MLT as described for glutamate or GABA [83]. As outlined above, the metabolites regulate the functional activity of MT receptors. Furthermore, the overlapping of pinealocytes and astrocytes could potentially prevent MLT from accessing its receptors. This behaviour would probably be dependent on environmental and physiological conditions such as circadian rhythm (day-night) or seasonal conditions (oestrus-anoestrus). Finally, the functional expression of MT receptors in astrocytes has been suggested since the mRNA encoding the MT2 receptor is expressed in astrocytes from chick diencephalon [84] and cerebellar Bergmann [85, 86].

In conclusion, our results demonstrate the presence of MT_1_ and MT_2_ receptors in pineal gland. They also present evidence for a negative feedback regulation of melatonin on its own synthesis. MT receptors counteract the action of adrenergic receptors to fine-tune MLT secretion by the pineal gland. Further study is necessary to relate this characteristic with the circadian and seasonal rhythm of melatonin synthesis.

## Supporting information

S1 Fig Effect of pineal MLT on binding properties of MT_1_ and MT_2_ receptors. Saturation curves were performed with 2-[^125I^]-MLT at 37°C for 1 h on ovine pineal gland cells preteated with 5 nM tryptophan hydroxylase inhibitor (PCPA) and 1 μM reserpine (triangle) or washed many times with HBSS before the experiments (circle). Specific binding is represented. The experiments were performed in duplicates and were repeated 2–3 times.
(TIF)

S2 Fig Expression kinetics of phospho-ERK in the presence of 10^−7^ M MLT. The Western blot is a representative experiment (B) and the curve (A) represent mean ± s.e.m. values of data obtained from duplicate in 3–5 different experiments *P<0.05 in Kruskal-Wallis tests followed by the Dunn’s multiple comparison tests.
(TIF)

S1 File (ZIP)

## References

[1] Reiter RJ, Tan D, Terron MP, Flores LJ, Czarnocki Z. Melatonin and its metabolites: new findings regarding their production and their radical scavenging actions. Acta Biochim Pol. 2007;54(1):1‑9. 17351668

[2] Shiu SYW, Pang B, Tam CW, Yao KM. Signal transduction of receptor-mediated antiproliferative action of melatonin on human prostate epithelial cells involves dual activation of Gαs and Gαq proteins: melatonin signaling in cell proliferation. J Pineal Res. 2010;49(3):301‑11. doi: 10.1111/j.1600-079X.2010.00795.x 20695976

[3] Guesdon V, Malpaux B, Delagrange P, Spedding M, Cornilleau F, Chesneau D, et al. Rapid effects of melatonin on hormonal and behavioral stressful responses in ewes. Psychoneuroendocrinology. 2013;38(8):1426‑34. doi: 10.1016/j.psyneuen.2012.12.011 23337408

[4] Liu J, Clough SJ, Hutchinson AJ, Adamah-Biassi EB, Popovska-Gorevski M, Dubocovich ML. MT1 and MT2 melatonin receptors: a therapeutic perspective. Annu Rev Pharmacol Toxicol. 2016;56(1):361‑83. doi: 10.1146/annurev-pharmtox-010814-124742 26514204 PMC5091650

[5] Cipolla-Neto J, Amaral FGD. Melatonin as a hormone: new physiological and clinical insights. Endocr Rev. 2018;39(6):990‑1028. doi: 10.1210/er.2018-00084 30215696

[6] Li T, Jiang S, Han M, Yang Z, Lv J, Deng C, et al. Exogenous melatonin as a treatment for secondary sleep disorders: a systematic review and meta-analysis. ‎ Front Neuroendocrinol. 2019;52:22‑8. doi: 10.1016/j.yfrne.2018.06.004 29908879

[7] Arendt J. Melatonin: countering chaotic time cues. Front Endocrinol. 2019;10(391):1‑16. doi: 10.3389/fendo.2019.00391 31379733 PMC6646716

[8] Saptadip S. Physiological and pharmacological perspectives of melatonin. Arch Physiol Biochem. 2020;10:1‑22. 10.1080/13813455.2020.1770799 32520581

[9] Acuña-Castroviejo D, Escames G, Venegas C, Díaz-Casado ME, Lima-Cabello E, López LC, et al. Extrapineal melatonin: sources, regulation, and potential functions. Cell Mol Life Sci. 2014;71(16):2997‑3025. doi: 10.1007/s00018-014-1579-2 24554058 PMC11113552

[10] Vivid D, Bentley G. Seasonal reproduction in vertebrates: melatonin synthesis, binding, and functionality using Tinbergen’s four questions. Molecules. 2018;23(3):(652) 1–53. 10.3390/molecules23030652 PMC6017951 29534047

[11] Zhao D, Yu Y, Shen Y, Liu Q, Zhao Z, Sharma R, et al. Melatonin synthesis and function: evolutionary history in animals and plants. Front Endocrinol. 2019;10(249):1‑16. doi: 10.3389/fendo.2019.00249 31057485 PMC6481276

[12] Reppert SM, Weaver DR, Ebisawa T. Cloning and characterization of a mammalian melatonin receptor that mediates reproductive and circadian responses. Neuron. 1994;13(5):1177‑85. doi: 10.1016/0896-6273(94)90055-8 7946354

[13] Reppert SM, Gusella JF. Molecular characterization of a second melatonin receptor expressed in human retina and brain: the Mel1b melatonin receptor. Proc Natl Acad Sci USA. 1995;92(19):8734‑8. doi: 10.1073/pnas.92.19.8734 7568007 PMC41041

[14] Dubocovich ML. Pharmacology and function of melatonin receptors. FASEB J. 1988;2(12):2765‑73. doi: 10.1096/fasebj.2.12.2842214 2842214

[15] Legros C, Devavry S, Caignard S, Tessier C, Delagrange P, Ouvry C, et al. Melatonin MT1 and MT2 receptors display different molecular pharmacologies only in the G-protein coupled state. Br J Pharmacol. 2014;171(1):186‑201. doi: 10.1111/bph.12457 24117008 PMC3874706

[16] Legros C, Brasseur C, Delagrange P, Ducrot P, Nosjean O, Boutin JA. Alternative radioligands for investigating the molecular pharmacology of melatonin receptors. J Pharmacol Exp Ther. 2016;356(3):681‑92. doi: 10.1124/jpet.115.229989 26759496

[17] Nosjean O, Ferro M, Cogé F, Beauverger P, Henlin JM, Lefoulon F, et al. Identification of the melatonin binding site MT3 as the quinone reductase 2. J Biol Chem. 2000;275(40):31311‑7. doi: 10.1074/jbc.M005141200 10913150

[18] Boutin JA, Ferry G. Is there sufficient evidence that the melatonin binding site MT3 is quinone reductase 2? J Pharmacol Exp Ther. 2019;368(1):59‑65. doi: 10.1124/jpet.118.253260 30389722

[19] Dubocovich ML, Delagrange P, Krause DN, Sugden D, Cardinali DP, Olcese J. International union of basic and clinical pharmacology. LXXV. nomenclature, classification, and pharmacology of G protein-coupled melatonin receptors. Pharmacol Rev. 2010;62(3):343‑80. doi: 10.1124/pr.110.002832 20605968 PMC2964901

[20] Jockers R, Delagrange P, Dubocovich ML, Markus RP, Renault N, Tosini G, et al. Update on melatonin receptors: IUPHAR Review 20: Melatonin receptors. Br J Pharmacol. 2016;173(18):2702‑25. doi: 10.1111/bph.13536 27314810 PMC4995287

[21] Cecon E, Oishi A, Jockers R. Melatonin receptors: molecular pharmacology and signalling in the context of system bias: melatonin receptor system bias. Br J Pharmacol. 2018;175(16):3263‑80. doi: 10.1111/bph.13950 28707298 PMC6057902

[22] De Reviers MM, Ravault JP, Tillet Y, Pelletier J. Melatonin binding sites in the sheep pars tuberalis. Neurosci Lett. 1989;100(1‑3):89‑93. doi: 10.1016/0304-3940(89)90665-4 2548131

[23] Morgan PJ, Williams LM, Davidson G, Lawson W, Howell E. Melatonin receptors on ovine pars tuberalis: characterization and autoradiographicai localization. J Neuroendocrinol. 1989;1(1):1‑4. doi: 10.1111/j.1365-2826.1989.tb00068.x 19210474

[24] Morgan PJ, King TP, Lawson W, Slater D, Davidson G. Ultrastructure of melatonin-responsive cells in the ovine pars tuberalis. Cell Tissue Res. 1991;263(3):529‑34. doi: 10.1007/BF00327285 1652362

[25] Morgan PJ, Barrett P, Davidson G, Lawson W. Melatonin regulates the synthesis and secretion of several proteins by pars tuberalis cells of the ovine pituitary. J Neuroendocrinol. 1992;4(5):557‑63. doi: 10.1111/j.1365-2826.1992.tb00204.x 21554640

[26] Gauer F, Masson-Pévet M, Pévet P. Melatonin receptor density is regulated in rat pars tuberalis and suprachiasmatic nuclei by melatonin itself. Brain Res. 1993;602(1):153‑6. doi: 10.1016/0006-8993(93)90256-m 8383569

[27] McNulty S, Rosst AW, Barrettt P, Hastings MH, Morgan PJ. Melatonin regulates the phosphorylation of CREB in ovine pars tuberalis. J Neuroendocrinol. 1994;6(5):523‑32. doi: 10.1111/j.1365-2826.1994.tb00615.x 7827622

[28] McNulty S, Ross AW, Shiu KY, Morgan PJ, Hastings MH. Phosphorylation of CREB in ovine pars tuberalis is regulated both by cyclic AMP-dependent and cyclic AMP-independent mechanisms. J Neuroendocrinol. 1996;8(8):635‑45. 8866252

[29] Malpaux B, Daveau AS, Maurice-Mandon FO, Duarte G, Chemineau P. Evidence that melatonin acts in the premammillary hypothalamic area to control reproduction in the ewe: presence of binding sites and stimulation of luteinizing hormone secretion by in situ microimplant delivery. Endocrinology. 1998;139(4):1508‑16. doi: 10.1210/endo.139.4.5879 9528928

[30] Migaud M, Daveau A, Malpaux B. MTNR1a melatonin receptors in the ovine premammillary hypothalamus: day-night variation in the expression of the transcripts. Biol Reprod. 2005;72(2):393‑8. doi: 10.1095/biolreprod.104.030064 15470001

[31] Malpaux B. Seasonal regulation of reproduction in mammals. In: Knobil and Neill’s Physiology of Reproduction. 2006. p. 2231‑81.

[32] Korf HW. Signaling pathways to and from the hypophysial pars tuberalis, an important center for the control of seasonal rhythms. Gen Comp Endocrinol. 2018;258:236‑43. doi: 10.1016/j.ygcen.2017.05.011 28511899

[33] Cogé F, Guenin S, Fery I, Migaud M, Devavry S, Slugocki C, et al. The end of a myth: cloning and characterization of the ovine melatonin MT2 receptor: Cloning and characterization of ovine MT2. Br J Pharmacol. 2009;158(5):1248‑62. doi: 10.1111/j.1476-5381.2009.00453.x 19814723 PMC2782334

[34] Stehle JH, Saade A, Rawashdeh O, Ackermann K, Jilg A, Sebestény T, et al. A survey of molecular details in the human pineal gland in the light of phylogeny, structure, function and chronobiological diseases: molecular details in the human pineal gland. J Pineal Res. 2011;51(1):17‑43. doi: 10.1111/j.1600-079X.2011.00856.x 21517957

[35] Rollag M, Niswender GD. Radioimmunoassay of serum concentrations of melatonin in sheep exposed to different lighting regimens. Endocrinology. 1976;482‑9. doi: 10.1210/endo-98-2-482 1248456

[36] Rollag MD, O’Callaghan PL, Niswender GD. Serum melatonin concentrations during different stages of the annual reproductive cycle in ewes. Biol Reprod. 1978;18(2):279‑85. doi: 10.1095/biolreprod18.2.279 564717

[37] Tillet Y, Ravault JP, Selve C, Evin G, Castro B, Dubois MP. Conditions for the use of specific antibodies for immunohistochemical visualization of serotonin and melatonin in the pineal gland of sheep. C R Acad Sci III. 1986;77‑82. 3093008

[38] Arendt J. Melatonin and the mammalian pineal gland. First Edition. CHAPMAN & HALL; 1995.

[39] Gómez Brunet A, Malpaux B, Daveau A, Taragnat C, Chemineau P. Genetic variability in melatonin secretion originates in the number of pinealocytes in sheep. J Endocrinol. 2002;172(2):397‑404. doi: 10.1677/joe.0.1720397 11834457

[40] Vakkuri O, Lamsa E, Rahkamaa E, Ruotsalainen H, Leppaluoto J. Lodinated melatonin: preparation and characterization of the molecular structure by mass and 1H NMR spectroscopy. Anal Biochem. 1984;142(2):284‑9. doi: 10.1016/0003-2697(84)90466-4 6549370

[41] Vanecek J, Pavlik A, Illnerova H. Hypothalarnic melatonin receptor sites revealed by autoradiography. Brain Res. 1987;435(1‑2):359‑62. 2827856 10.1016/0006-8993(87)91625-8

[42] Legros C, Matthey U, Grelak T, Pedragona-Moreau S, Hassler W, Yous S, et al. New radioligands for describing the molecular pharmacology of MT1 and MT2 melatonin receptors. Int J Mol Sci. 2013;14(5):8948‑62. doi: 10.3390/ijms14058948 23698757 PMC3676766

[43] Chan ASL, Lai FPL, Lo RKH, Voyno-Yasenetskaya TA, Stanbridge EJ, Wong YH. Melatonin MT1 and MT2 receptors stimulate c-Jun N-terminal kinase via pertussis toxin-sensitive and -insensitive G proteins. ‎Cell signalling. 2002;14(3):249‑57. doi: 10.1016/s0898-6568(01)00240-6 11812653

[44] Guillaume JL, Daulat AM, Maurice P, Levoye A, Migaud M, Brydon L, et al. The PDZ protein Mupp1 promotes Gi coupling and signaling of the MT 1 melatonin receptor. J Biol Chem. 2008;283(24):16762‑71. doi: 10.1074/jbc.M802069200 18378672

[45] Maurice P, Daulat AM, Turecek R, Ivankova-Susankova K, Zamponi F, Kamal M, et al. Molecular organization and dynamics of the melatonin MT1 receptor/RGS20/Gi protein complex reveal asymmetry of receptor dimers for RGS and Gi coupling. EMBO J. 2010;29(21):3646‑59. doi: 10.1038/emboj.2010.236 20859254 PMC2982759

[46] Chen M, Cecon E, Karamitri A, Gao W, Gerbier R, Ahmad R, et al. Melatonin MT1 and MT2 receptor ERK signaling is differentially dependent on Gi/o and Gq/11 proteins. J Pineal Res. 2020;68(4):1‑12. doi: 10.1111/jpi.12641 32080899

[47] Dubocovich ML, Masana MI, Iacob S, Sauri DM. Melatonin receptor antagonists that differentiate between the human Mel1a and Mel1b recombinant subtypes are used to assess the pharmacological profile of the rabbit retina ML1 presynaptic heteroreceptor: Naunyn-Schmiedeberg’s Arch Pharmacol. 1997;355(3):365‑75. doi: 10.1007/pl00004956 9089668

[48] Dubocovich ML, Yun K, Al‐Ghoul WM, Benloucif S, Masana MI. Selective MT2 melatonin receptor antagonists block melatonin-mediated phase advances of circadian rhythms. FASEB J. 1998;12(12):1211‑20. doi: 10.1096/fasebj.12.12.1211 9737724

[49] Browning C, Beresford I, Fraser N, Giles H. Pharmacological characterization of human recombinant melatonin MT1 and MT2 receptors. Br J Pharmacol. 2000;129(5):877‑86. doi: 10.1038/sj.bjp.0703130 10696085 PMC1571913

[50] Howell HE, Morgan PJ. beta-adrenergic stimulation increases cAMP and melatonin production in ovine pinealocyte cultures. J Pineal Res. 1991;10(3):122‑9. doi: 10.1111/j.1600-079x.1991.tb00828.x 1679132

[51] Gupta BB, Spessert R, Vollrath L. Molecular components and mechanism of adrenergic signal transduction in mammalian pineal gland: regulation of melatonin synthesis. Indian J Exp Biol. 2005;43(2):115‑49. 15782814

[52] Saha S, Singh KM, Gupta BBP. Melatonin synthesis and clock gene regulation in the pineal organ of teleost fish compared to mammals: similarities and differences. Gen Comp Endocrinol. 2019;279:27‑34. doi: 10.1016/j.ygcen.2018.07.010 30026020

[53] González S, Moreno-Delgado D, Moreno E, Pérez-Capote K, Franco R, Mallol J, et al. Circadian-related heteromerization of adrenergic and dopamine D4 receptors modulates melatonin synthesis and release in the pineal gland. PLoS Biol. 2012;10(6):e1001347. doi: 10.1371/journal.pbio.1001347 22723743 PMC3378626

[54] Lewczuk B, Ziółkowska N, Prusik M, Przybylska-Gornowicz B. Adrenergic activation of melatonin secretion in ovine pineal explants in short-term superfusion culture occurs via protein synthesis independent and dependent phenomena. Biomed Res. Int. 2014;2014:1‑11. doi: 10.1155/2014/715708 25133175 PMC4123513

[55] Bondi CD, McKeon RM, Bennett JM, Ignatius PF, Brydon L, Jockers R, et al. MT1 melatonin receptor internalization underlies melatonin-induced morphologic changes in chinese hamster ovary cells and these processes are dependent on Gi proteins, MEK 1/2 and microtubule modulation. J Pineal Res. 2008;44(3):288‑98. doi: 10.1111/j.1600-079X.2007.00525.x 18339124

[56] Sung JY, Bae JH, Lee JH, Kim YN, Kim DK. The melatonin signaling pathway in a long-term memory in vitro study. Molecules. 2018;23(4):1‑15. 10.3390/molecules23040737 PMC6017053 29570621

[57] Hazlerigg DG, Thompson M, Hastings MH, Morgan PJ. Regulation of mitogen-activated protein kinase in the pars tuberalis of the ovine pituitary: interactions between melatonin, insulin-like growth factor-l, and forskolin. Endocrinology. 1996;137(1):210‑8. doi: 10.1210/endo.137.1.8536615 8536615

[58] Trecherel E, Batailler M, Chesneau D, Delagrange P, Malpaux B, Chemineau P, et al. Functional characterization of polymorphic variants for ovine MT1 melatonin receptors: possible implication for seasonal reproduction in sheep. Anim Reprod Sci. 2010;122(3‑4):328‑34. doi: 10.1016/j.anireprosci.2010.10.007 21075566

[59] Audinot V, Bonnaud A, Grandcolas L, Rodriguez M, Nagel N, Galizzi JP, et al. Molecular cloning and pharmacological characterization of rat melatonin MT1 and MT2 receptors. Biochem Pharmacol. 2008;75(10):2007‑19. doi: 10.1016/j.bcp.2008.02.022 18384758

[60] Mailliet F, Audinot V, Malpaux B, Bonnaud A, Delagrange P, Migaud M, et al. Molecular pharmacology of the ovine melatonin receptor: comparison with recombinant human MT1 and MT2 receptors. Biochem Pharmacol. 2004;67(4):667‑77. doi: 10.1016/j.bcp.2003.09.037 14757166

[61] Privat K, Brisson C, Jouvet A, Chesneau D, Ravault J-P, Fevre-Montange M. Evidence for implication of tryptophan hydroxylase in the regulation of melatonin synthesis in ovine pinealocytes in culture. Cell Mol Neurobiol. 2002;13. doi: 10.1023/a:1015385527187 12507391 PMC11533740

[62] Namboodiri M, Sugden D, Klein DC, Tamarkin L, Mefford N. Serum melatonin and pineal indoleamine metabolism in a species with a small day/night N-Acetyltransferase rhythm. Comp Biochem Physiol. 1985;80B(4):731‑6. 10.1016/0305-0491(85)90453-5 3995918

[63] Namboodiri MA, Sugden D, Klein DC, Grady R, Mefford IN. Rapid nocturnal increase in ovine pineal N-Acetyltransferase activity and melatonin synthesis: effects of cycloheximide. J Neurochem. 1985;45(3):832‑5. doi: 10.1111/j.1471-4159.1985.tb04069.x 2411856

[64] Sugden D, Namboodiri MAA, Klein DC, Grady RK, Mefford IN. Ovine pineal indoles: effects of L-tryptophan or L-5-hydroxytryptophan administration. J Neurochem. 1985;44(3):769‑72. doi: 10.1111/j.1471-4159.1985.tb12881.x 3871838

[65] Ravault JP, Chesneau D, Ouvray M, Locatelli A. Pineal microdialysis of the melatonin in conscious sheep: methodology, application to a diurnal rhythm and effect of isoproterenol. J Neuroendocrinol. 1996;8(5):387‑94. doi: 10.1046/j.1365-2826.1996.04667.x 8736438

[66] Maronde E, Saade A, Ackermann K, Goubran-Botros H, Pagan C, Bux R, et al. Dynamics in enzymatic protein complexes offer a novel principle for the regulation of melatonin synthesis in the human pineal gland: novel regulatory principles in human melatonin synthesis. J Pineal Res. 2011;51(1):145‑55. doi: 10.1111/j.1600-079X.2011.00880.x 21517958

[67] Ganguly S, Weller JL, Ho A, Chemineau P, Malpaux B, Klein DC. Melatonin synthesis: 14-3-3-dependent activation and inhibition of Arylalkylamine N-Acetyltransferase mediated by phosphoserine-205. Proc Natl Acad Sci USA. 2005;102(4):1222‑7. doi: 10.1073/pnas.0406871102 15644438 PMC544185

[68] Schomerus C, Laedtke E, Korf HW. Activation of Arylalkylamine N-Acetyltransferase by phorbol esters in bovine pinealocytes suggests a novel regulatory pathway in melatonin synthesis. J Neuroendocrinol. 2004;16(9):741‑9. doi: 10.1111/j.1365-2826.2004.01228.x 15344912

[69] Barbosa R, Helena Scialfa J, Mingarini Terra I, Cipolla-Neto J, Simonneaux V, Castro Afeche S. Tryptophan hydroxylase is modulated by L-type calcium channels in the rat pineal gland. Life Sciences. 2008;82(9‑10):529‑35. doi: 10.1016/j.lfs.2007.12.011 18221757

[70] Afeche SC, Barbosa R, Scialfa JH, Terra IM, Cassola AC, Cipolla-Neto J. Effects of the blockade of high voltage-activated calcium channels onin vitro pineal melatonin synthesis. Cell Biochem Funct. 2006;24(6):499‑505. doi: 10.1002/cbf.1270 16143961

[71] Yamada H, Hayashi M, Uehara S, Kinoshita M, Muroyama A, Watanabe M, et al. Norepinephrine triggers Ca2+-dependent exocytosis of 5-hydroxytryptamine from rat pinealocytes in culture: Exocytosis of 5-HT from rat pinealocytes. J Neurochem. 2002;81(3):533‑40. doi: 10.1046/j.1471-4159.2002.00839.x 12065661

[72] Yamada H, Ogura A, Koizumi S, Yamaguchi A, Moriyama Y. Acetylcholine triggers L-glutamate exocytosis via nicotinic receptors and inhibits melatonin synthesis in rat pinealocytes. J Neurosci. 1998;18(13):4946‑52. doi: 10.1523/JNEUROSCI.18-13-04946.1998 9634560 PMC6792550

[73] Yamada H, Yatsushiro S, Ishio S, Hayashi M, Nishi T, Yamamoto A, et al. Metabotropic glutamate receptors negatively regulate melatonin synthesis in rat pinealocytes. J Neurosci. 1998;18(6):2056‑62. doi: 10.1523/JNEUROSCI.18-06-02056.1998 9482792 PMC6792920

[74] Møller M, Baeres FM. The anatomy and innervation of the mammalian pineal gland. Cell Tissue Res. 2002;309(1):139‑50. doi: 10.1007/s00441-002-0580-5 12111544

[75] Mays JC, Kelly MC, Coon SL, Holtzclaw L, Rath MF, Kelley MW, et al. Single-cell RNA sequencing of the mammalian pineal gland identifies two pinealocyte subtypes and cell type-specific daily patterns of gene expression. PLoS ONE. 2018;13(10):1‑22. doi: 10.1371/journal.pone.0205883 30347410 PMC6197868

[76] Rath MF, Coon SL, Amaral FG, Weller JL, Møller M, Klein DC. Melatonin synthesis: Acetylserotonin o-Methyltransferase (ASMT) is strongly expressed in a subpopulation of pinealocytes in the male rat pineal gland. Endocrinology. 2016;157(5):2028‑40. doi: 10.1210/en.2015-1888 26950199 PMC4870883

[77] Desagher S, Glowinski J, Premont J. Astrocytes protect neurons from hydrogen peroxide toxicity. J Neurosci. 1996;16(8):2553‑62. doi: 10.1523/JNEUROSCI.16-08-02553.1996 8786431 PMC6578753

[78] Araque A, Carmignoto G, Haydon PG. Dynamic signaling between astrocytes and neurons. Annu Rev Physiol. 2001;63(1):795‑813. doi: 10.1146/annurev.physiol.63.1.795 11181976

[79] Sofroniew MV. Reactive astrocytes in neural repair and protection. Neuroscientist. 2005;11(5):400‑7. doi: 10.1177/1073858405278321 16151042

[80] González J, Pinzón A, Angarita-Rodríguez A, Aristizabal AF, Barreto GE, Martín-Jiménez C. Advances in astrocyte computational models: from metabolic reconstructions to multi-omic approaches. Front Neuroinform. 2020;14(35):1‑13. doi: 10.3389/fninf.2020.00035 32848690 PMC7426703

[81] Zang X, Nilaver G, Stein BM, Michael R. Fetell MR, Duffy PE. Immunocytochemistry of pineal astrocytes: species differences and functional implications. J Neuropathol Exp Neurol. 1985;44(5):486‑95. doi: 10.1097/00005072-198509000-00004 3897467

[82] Villela D, Atherino VF, Lima L de S, Moutinho AA, Amaral FGD, Peres R, et al. Modulation of pineal melatonin synthesis by glutamate involves paracrine interactions between pinealocytes and astrocytes through NF- κ B activation. Biomed Res Int. 2013;2013:1‑14. 10.1155/2013/618432 PMC3747608 23984387

[83] Hertz L, Rodrigues TB, éditeurs. Astrocytic-neuronal-astrocytic pathway selection for formation and degradation of glutamate/GABA. Front Endocrinol. 2014;5(42):1‑4. doi: 10.3389/fendo.2014.00042 24772106 PMC3982103

[84] Adachi A, Natesan AK, Whitfield-Rucker MG, Weigum SE, Cassone VM. Functional melatonin receptors and metabolic coupling in cultured chick astrocytes. Glia. 2002;39(3):268‑78. doi: 10.1002/glia.10109 12203393

[85] Al-Ghoul WM, Herman MD, Dubocovich ML. Melatonin receptor subtype expression in human cerebellum. NeuroReport. 1998;9(18):4063‑8. doi: 10.1097/00001756-199812210-00011 9926848

[86] López-Bayghen E, Rosas S, Castelán F, Ortega A. Cerebellar Bergmann glia: an important model to study neuron-glia interactions. Neuron Glia Biol. 2007;3(2):155‑67. doi: 10.1017/S1740925X0700066X 18634572

