# Supplementary figures and images for: Negative regulation of melatonin secretion by melatonin receptors in ovine pinealocytes
Source: PLoS One. 2021 Jul 29;16(7):e0255249. doi: 10.1371/journal.pone.0255249 (PMC8320996; doi:10.1371/journal.pone.0255249)

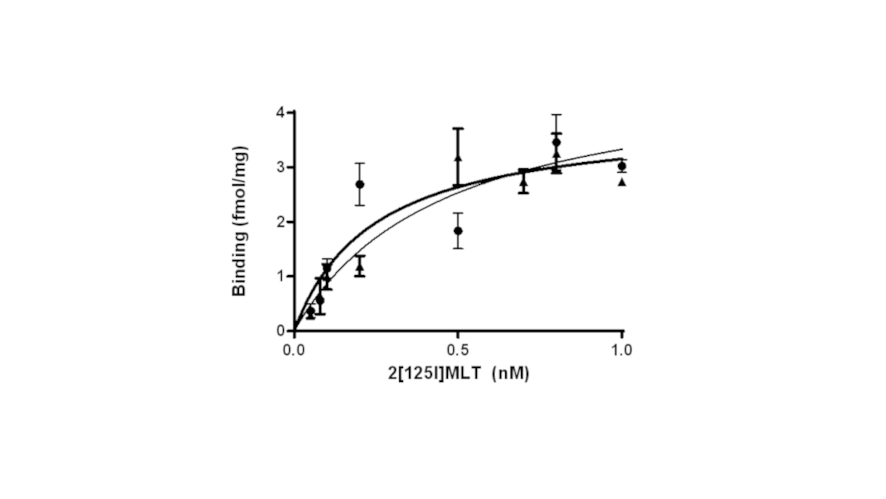

Supplement: S1 Fig — Saturation curves were performed with 2-[125I]-MLT at 37°C for 1 h on ovine pineal gland cells preteated with 5 nM tryptophan hydroxylase inhibitor (PCPA) and 1 μM reserpine (triangle) or washed many times with HBSS before the experiments (circle). Specific binding is represented. The experiments were performed in duplicates and were repeated 2–3 times. (TIF) [file pone.0255249.s001.tif]

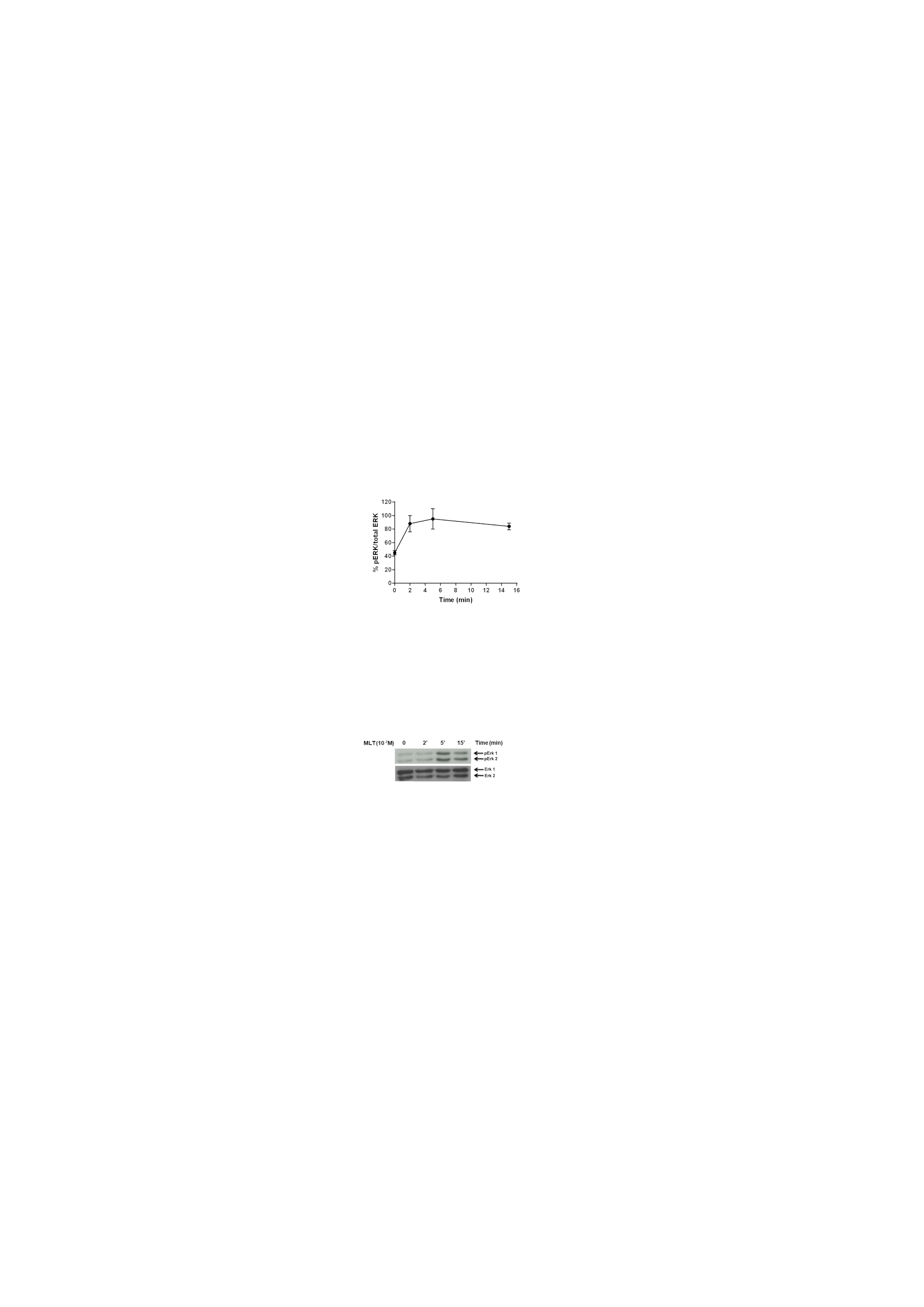

Supplement: S2 Fig — The Western blot is a representative experiment (B) and the curve (A) represent mean ± s.e.m. values of data obtained from duplicate in 3–5 different experiments *P<0.05 in Kruskal-Wallis tests followed by the Dunn’s multiple comparison tests. (TIF) [file pone.0255249.s002.tif]

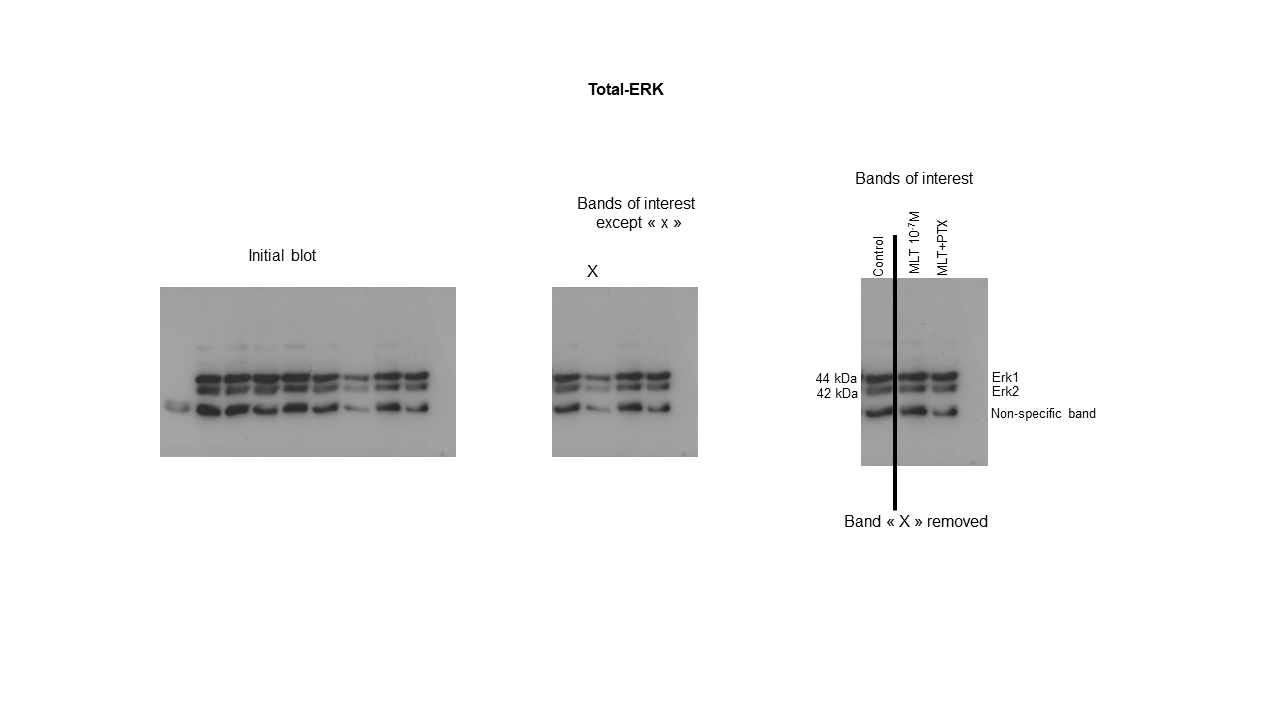

Supplement: S1 File — (ZIP) [file pone.0255249.s003.zip › Original Fig 3C-ERK.tif]

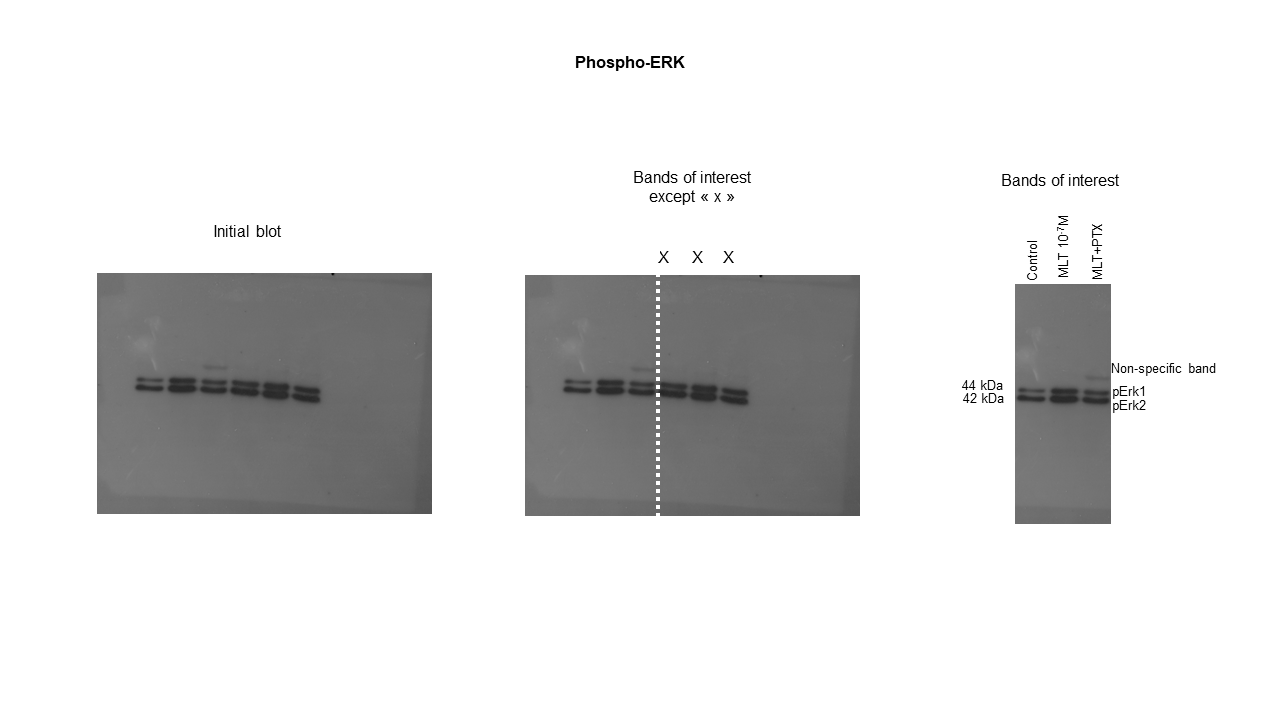

Supplement: S1 File — (ZIP) [file pone.0255249.s003.zip › Original Fig 3C-pERK.tif]

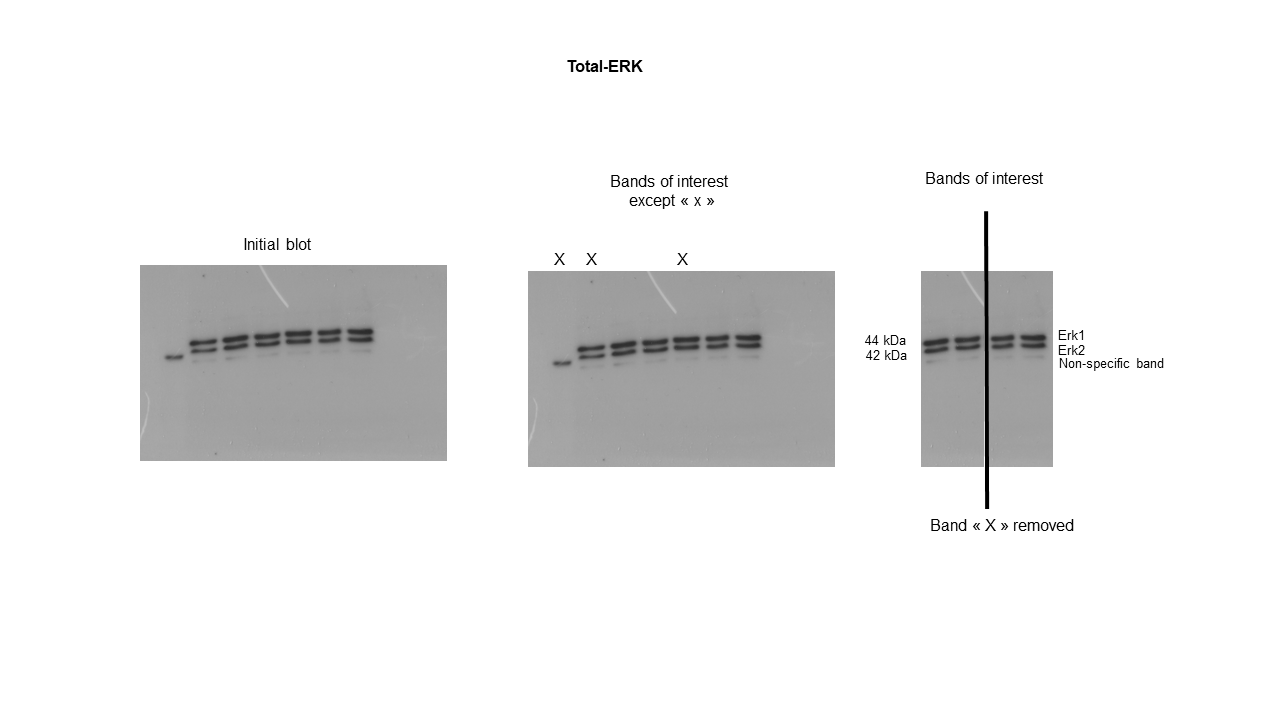

Supplement: S1 File — (ZIP) [file pone.0255249.s003.zip › Original S2B Fig-ERK.tif]

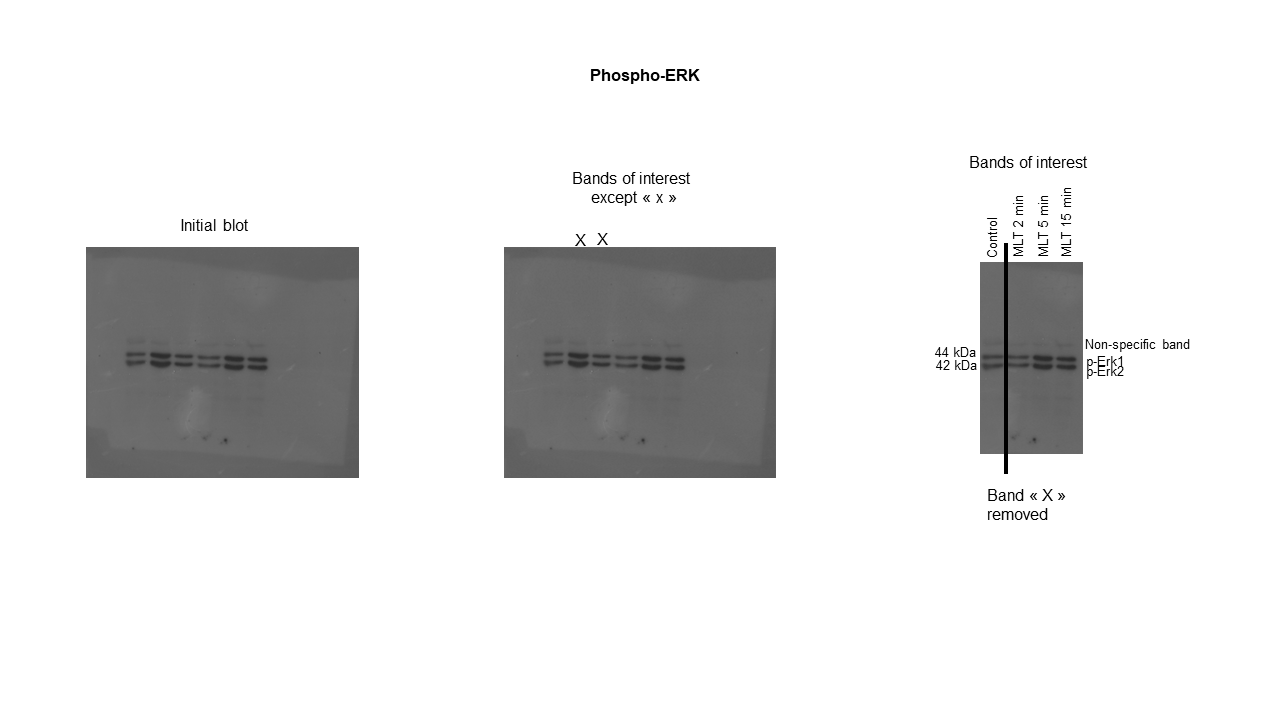

Supplement: S1 File — (ZIP) [file pone.0255249.s003.zip › Original S2B Fig-pERK.tif]
